# Demonstration of Competence in the Bachelor of Physiotherapy Degree – Qualitative Study of Physiotherapy Students’ Experiences of a Competence-Based Assessment

**DOI:** 10.1177/23821205261446858

**Published:** 2026-04-25

**Authors:** Tuomainen Paula, Äijö Marja, Nieminen Heidi, Haaranen Ari

**Affiliations:** 1 4345 School of Health Care, Savonia University of Applied Sciences, Kuopio, Finland; 2 Department of Physical and Rehabilitation Medicine, Kuopio University Hospital, Kuopio, Finland; 3 Department of Nursing Science, University of Eastern Finland, Kuopio, Finland

**Keywords:** physiotherapy education, competency-based assessment, student experience, qualitative research, professional competence

## Abstract

**Background:**

Practical skill assessment is essential in health and social care education to ensure students are prepared for real-world clinical situations. Competency-based assessments (CBA) offer a structured way to evaluate students’ psychomotor, affective, and cognitive skills. While widely used in nursing education, their application in physiotherapy education remains limited.

**Objective:**

This study aimed to explore physiotherapy students’ experiences of the CBA conducted at a University of Applied Sciences.

**Methods:**

A qualitative research design was employed. Data were collected through semi-structured focus-group interviews with 28 students and analyzed using inductive thematic analysis.

**Results:**

Physiotherapy students perceived the assessment as realistic and effective in measuring competence. It enabled practical application of theoretical knowledge and enhanced professional confidence. Although initial anxiety was common, a supportive atmosphere and post-assessment evaluation discussion promoted learning. Students valued the opportunity to demonstrate their skills in a setting that resembled authentic physiotherapy practice.

**Conclusion:**

CBA can support learning and professional development in physiotherapy education. This study highlights their potential to align educational practices with workplace demands. Future research should examine educators’ perspectives and the long-term impact of such assessments on graduates’ transition to working life.

## Introduction

Physiotherapy education aims to ensure that graduating professionals can work independently, delivering high-quality and evidence-based care.^1^ In Finland, Universities of Applied Sciences provide competence-based and practice-oriented education designed to meet the needs of working life.^2^ The curricula are built on competence-based frameworks, which align with the European Qualifications Framework.^3^ According to the Finnish Universities of Applied Sciences Act (932/2014), higher education must meet the demands of working life and equip students with the skills required for expert roles.^4^ Each institution has the autonomy to define the content of its curriculum, including the methods used to assess student competence.

As part of this goal, increasing emphasis is placed on practical competence and preparedness for clinical practice. One widely used method for evaluating clinical skills is competency-based assessment (CBA). It is a practical assessment in which students demonstrate mastery of essential knowledge and skills required in the physiotherapy profession, either in an authentic or simulated environment.^5^ Compared to traditional written examinations, the CBA allows for the assessment of psychomotor, cognitive, and emotional domains, and helps identify areas for improvement.^6^ The CBA used in this study most closely resembled an OSPE/OSCE structured practical examination, as students performed a single comprehensive clinical scenario that was observed and assessed using criteria-based rubrics.

Previous research has explored students’ experiences of CBA in various fields of health and social care, with most studies focusing on nursing education and only a few on physiotherapy. Overall, students have responded positively to these exams^7-11^ and considered them a useful learning method.^12-15^ According to students’ experiences, clear instructions provided in advance^7,8,11,14,16^ and sufficient time for preparation^14,15,17^ are essential for successful exam readiness. Students reported feelings of stress and anxiety both before^8,15,17,18^ and during the exam/assessment.^10,12,13,19,20^ Despite this, they were generally satisfied with the content of the assessment.^14,19^ The assessment helped students identify areas for improvement, which was seen as beneficial for learning.^10,11,14,17^ Transparent assessment practices based on clear criteria contributed to more positive attitudes toward the exam.^11,14^

While physiotherapy-specific literature has examined students’ perceptions of their competence,^21^ research focusing on physiotherapy students’ experiences of competency-based assessments remains limited. Existing studies highlight that perceived competence grows particularly in authentic clinical environments^21^ and that new graduates often face challenges related to increased workload and clinical complexity during the transition to professional practice.^22^ These findings emphasize the importance of understanding how physiotherapy students experience CBA within higher education settings. Although some international studies have explored physiotherapy students’ experiences of structured practical assessments, research remains limited, especially regarding CBA implementations within European higher education contexts.

This study was carried out in spring 2025, when CBA was introduced as part of the final-stage assessment of physiotherapy students’ skills in a University of Applied Sciences. The aim of this study was to explore physiotherapy students’ experiences of CBA conducted at a University of Applied Sciences.

## Methods

### Participants and Study Setting

The study involved 39 physiotherapy students from a single cohort at a University of Applied Sciences, all of whom participated in CBA in spring 2025. Interview inclusion criteria included participation in the CBA and consent to participate in a focus-group interview. Students who did not complete the CBA or declined participation from interview were excluded. From totally 28 students voluntarily participated in interviews conducted in five groups of 5–7 participants. The interviews lasted 12–52 minutes, with an average duration of 31 minutes. The transcribed material amounted to 72 pages (font: Open Sans, size: 11 pt, line spacing: 1.5; margins: 2 cm and 1.5 cm). The data were collected over one week between 16 and 22 May 2025.

### Competency-Based Assessment (CBA)

The two-part CBA was based on the professional competencies of physiotherapists.^23,24^ The development of the rubrics was grounded in the collaborative expertise of several physiotherapy professionals with extensive experience in both clinical practice and education. The theoretical component prior to the practical assessment included 68 multiple-choice and statement-based questions that broadly evaluated students’ knowledge of core physiotherapy topics and previously completed study modules.

Two weeks prior to the practical part of the assessment, students received information outlining the assessment structure and case topics. In the assessment, one teacher acted as the client while another evaluated performance using rubric based on physiotherapy competencies. The practical assessment involved 18 client cases, covering core areas of physiotherapy, respiratory and cardiovascular systems, neurology, and musculoskeletal care, across different stages of the physiotherapy process. Nearly all the cases used in the CBA had been previously piloted in teaching settings, either within simulation sessions or during supervised practice classes. Client cases were randomly assigned to students. After that the student had 15 minutes to prepare and 20–30 minutes to complete the performance. A 15-minute evaluation discussion followed, focusing on competence and learning. Assessments were conducted in simulated environments at the university, such as hospital wards, home settings, or consultation rooms. Each case specific assessment framework adheres to a consistent core competency evaluation, complemented by an assessment rubric tailored to the specific case. Both parts of the CBA were conducted using a pass–fail scale, with a passing threshold set at 60%.

### Data Collection

Prior to data collection, the participants were informed about the upcoming study via email. The researcher sent an invitation to participate along with an information sheet through a designated contact person. Before the interviews began, students had the opportunity to ask questions about the study, and they were also asked to submit a signed consent form.

Interviews served as the data collection method in this qualitative study, as the focus was on individuals’ experiences and thoughts.^25^ The data were collected through reflective learning discussions led by one researcher (PT), conducted in small groups on weekdays following the performance-based assessment. The interviews took place in a classroom at the University of Applied Sciences, with no teachers present during the discussions. The interviewer (PT) was a female Master of Health Sciences student and physiotherapist, with prior clinical and academic experience in physiotherapy. No prior relationship existed between the interviewer and participants. Participants were informed that the interviewer was a physiotherapy graduate student conducting the study as part of academic research. No personal details beyond the study purpose were provided.

**Figure 1.**
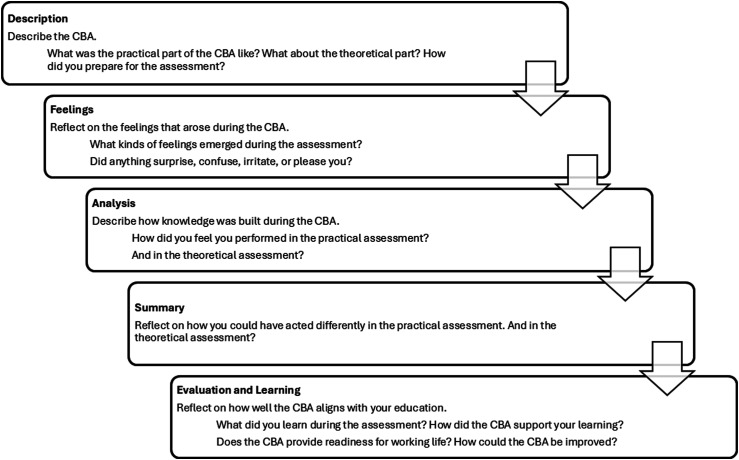
Semi-structured interview guide

The researcher utilized a semi-structured interview guide (Figure 1) specifically developed for this study, based on the learning discussion model which is grounded in Kolb’s experiential learning cycle.^26^ The model structured the learning discussion into four thematic stages and a final summary: personal experiences, emotions evoked by the assessment, analyzed how knowledge was constructed during the performance, and finally evaluated their own learning. Data saturation was reached during the focus-group interviews, as no new themes emerged in the later interviews.

## Data Analysis

The data were analyzed using inductive thematic analysis to identify themes emerging from the learning discussions that addressed the research questions and revealed deeper meanings in participants’ experiences and perspectives.^27^

The analysis followed six phases. First, the researcher immersed in the data by repeatedly listening to recordings and reading transcripts, making initial notes and observations.^28,29^ In the second phase, the dataset was systematically coded to identify relevant content, with concise analytical codes organized in Excel—initially by research question, then grouped by similarity or contrast.^27^

In phase three, codes were grouped into themes aligned with the research question, supported by theme maps to aid identification and validation.^30^ Phase four involved reviewing and refining the fit between themes, codes, and the original data, ensuring completeness and representativeness.^28,30^ In phase five, themes were named to reflect their core content and boundaries, with attention to whether they captured the essence of the data and addressed the main research question. Theme names were crafted to be concise, informative, and engaging.^28^

In the final phase, the analysis process was documented, and findings were compared with existing literature. The figures and tables support the presentation of the analytical process and the resulting themes. Selected anonymized excerpts from the original data were included to illustrate theme development, with dialectal and colloquial expressions edited to ensure confidentiality.^28^ Data saturation was reached during the focus-group interviews. An example of the thematic analysis process is shown in Table 1. The reporting of this qualitative study conforms to the Consolidated Criteria for Reporting Qualitative Research (COREQ) checklist.^31^

**Table 1.** Example of the Progression of Thematic Analysis

Original quote	Code	Subtheme	Main theme
It was very applied. I think you can’t just rely on memorizing bones, muscles and tests anymore. You have to think more holistically.	Integration and application of previously learned knowledge	Enhancing work-life readiness	Usefulness of CBA for the future career competencies
In this exam, I was able to combine things I had learned earlier. I noticed I could use what I had learned during internships.			
Now I have notes ready for situations like this. If I’m working in outpatient care or somewhere with frequent clients, I can quickly review before going in.	Practical tools for future situations		
It strengthened the idea that meeting the client is the most important thing and being present in that situation.	Importance of interaction	Supporting professional growth	
It reinforced the idea that you shouldn’t see the client only through their shoulder pain. It’s not just the condition — the person is the client, with a family, job, goals and dreams. That experience tied it all together.			
It strengthened the idea that meeting the client is the most important part and being present in the situation. And that physiotherapy can be done in your own style, as long as you’re doing the right things.	Using one’s personality in professional work		

## Results

Through thematic analysis, three main themes emerged from physiotherapy students’ experiences of the CBA: emotional experiences during the CBA, its role in supporting the learning process, and its perceived usefulness for developing future career competencies (Table 2).

**Table 2.** Description of Characteristics of the Theme

Characteristics	Theme
The CBA situation and its implementation evoked a range of emotional responses among students. While some experienced heightened emotions such as nervousness or surprise, others reported feeling neutral.	Emotional experience of CBA
Students described how the CBA supported their learning at multiple points in the process. The learning process included preparation, learning environment, and post-assessment evaluation discussion.	CBA enabling learning process
Students perceived the CBA as beneficial for their future professional development. Work-life readiness and professional growth were frequently highlighted.	Usefulness of CBA for the future career competencies

**Figure 2.**
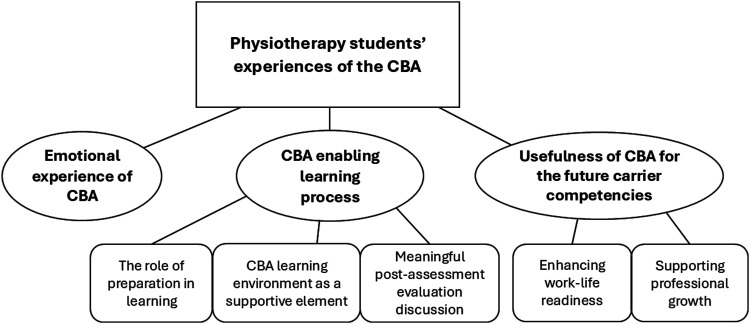
Physiotherapy students’ experiences of the CBA

These themes, illustrated in Figure 2, encompass emotional experience of CBA, CBA enabling the learning process, and usefulness of CBA for future career competencies. Although the demonstration exam included both practical and theoretical components, students’ interview reflections focused primarily on the practical part, likely due to the familiarity of the theoretical exam format.

## Emotional Experience of CBA

Physiotherapy students’ experiences of the CBA situation were mostly positive, and the CBA was perceived as supportive of learning. Many students described feeling intense nervousness before the exam. This nervousness began several months in advance and manifested not only as mental pressure but also through physical reactions. However, once the CBA began, the nervousness typically eased, allowing students to focus on their performance. The anxiety was often fueled by negative preconceptions and imagined scenarios about the upcoming situation. Frequently, the nervousness was linked to uncertainty about one’s competence and whether it would be sufficient in the CBA setting. In retrospect, some students felt their anxiety had been unnecessary.

“At first I was extremely nervous, but once I got started, I didn’t have time to be nervous anymore.” (Student 10)

“Then I went there to prepare, and I started to feel like I actually knew something. I decided to just go with whatever came to mind. We were allowed to use our notes, and there was time to go through them calmly.” (Student 19)

Some students approached the CBA with a neutral attitude and did not feel nervous. They saw that the CBA would test their realistic competence and felt they had already learned the necessary skills during their studies.

“I wasn’t nervous at all.” (Student 13)

“I didn’t really feel nervous. I just went in and did what I knew.” (Student 16)

Additionally, students were surprised by the relaxed atmosphere of the CBA. Many had expected a high-pressure situation with strict evaluation, like previous practical exams.

“I was surprised there weren’t any tricky questions, and it was all quite general.” (Student 13)

“I was surprised by how relaxed the situation was. I had been nervous that I’d be under pressure for half an hour.” (Student 3

On the other hand, some students found the situation challenging because the “client” was a familiar teacher. This made it difficult to fully adopt a professional role and, for some, even caused amusement.

“I kept laughing at the teacher’s acting… it was kind of distracting.” (Student 18)

## CBA Enabling Learning Process

The CBA supported learning throughout its various stages. Thematic analysis revealed three key aspects contributing to this: students’ own preparation, the learning environment, and the post-assessment evaluation discussion.

### The Role of Preparation in Learning

Most students prepared for the practical part using their personal learning styles. Preparation included reviewing course materials and notes, gathering theoretical knowledge relevant to client cases, and practicing practical skills individually or with peers. Some focused-on topics they found challenging, while others struggled with the broad scope of content and were unsure where to concentrate on their efforts. A few students chose not to prepare at all, viewing the CBA as a way to test their actual competence.

“My notes covered a bit of everything. I could have prepared more, but I trusted that I’d remember what I’ve learned over the past three years.” (Student 20)

“I read a lot of theory and tried to imagine what kinds of cases might come up.” (Student 4)

“We practiced together and discussed possible tests and client scenarios.” (Student 7)

Students generally did not prepare specifically for the theoretical part, feeling that their education had equipped them well. Some briefly reviewed course materials, while others relied entirely on their prior learning.

“I didn’t study at all. I figured the things I’ve learned over three years would come back and they did.” (Student 7)

“I saw this as a test of my actual level. I felt my studies had prepared me for it.” (Student 14)

### CBA Learning Environment as a Supportive Element

Students described the organization of the CBA as successful, highlighting the relaxed atmosphere and the authenticity of the client interaction. These impressions were shaped by the teacher’s role performance, the carefully constructed environment, and the teachers’ presence and attitude. The atmosphere was described as calm, gentle, and safe, allowing students to focus without interruptions.

“There was no rush, which created a safe feeling… even though I was nervous, I felt like everything was okay.” (Student 2)

“I liked that I could work calmly. The situation wasn’t too intense.” (Student 26)

The CBA required both students and teachers to fully engage in their roles. Many praised the teachers’ performances, saying it helped them immerse themselves in the situation and even forget they were being assessed. However, some felt that having a familiar teacher as the client reduced the authenticity. Challenges also arose when the CBA setting or equipment was unfamiliar.

“The teacher really committed to the role, which made it easier to understand the situation and the type of client.” (Student 28)

“It might have been easier with a real client, but the teacher played the role well enough.” (Student 16)

“The teacher in the client role was so convincing that I forgot someone was observing me.” (Student 1)

### Meaningful Post-assessment Evaluation Discussion

The post-assessment evaluation discussion was seen as a meaningful part of the learning and assessment process. It allowed students to reflect on their performance and review the CBA events. Students appreciated receiving immediate feedback and learning whether they had passed.

Positive feedback helped students recognize their strengths, which they might not have noticed themselves. Many tend to focus on mistakes, so encouraging feedback helped balance their self-assessment. Teachers also gave students space to share their own reflections, deepening the learning experience. The discussion was especially valuable when no grade was given, and some students wished for more time to explore details. Personalized feedback was considered particularly impactful.

“The debrief was helpful… I tend to focus on what went wrong, but the teachers also pointed out what went well.” (Student 3)

“At first, I felt awkward and stiff. But after getting feedback and reflecting, I realized it went quite well.” (Student 2)

“In an exam without a grade, the feedback discussion is crucial. It could have had as much time as the exam itself, personal feedback sticks better.” (Student 18)

## Usefulness of CBA for the Future Career Competencies

This main theme consists of two subthemes: the CBA’s role in developing work-life readiness and its contribution to professional growth.

### Enhancing work-life readiness

Nearly all students felt that the CBA provided concrete skills for working life. It was seen as an effective way to assess and validate competence before entering the profession, both from the student’s and educator’s perspective. The CBA offered an opportunity to apply previously learned knowledge in practice and to evaluate one’s skills more holistically.

“You got to show your own way of working, even though the client case was quite limited.” (Student 15)

“I noticed I could use things I had learned during internships.” (Student 17)

While some students felt they didn’t learn anything entirely new during the CBA itself, they all agreed that the review and repetition were beneficial. The CBA encouraged revisiting earlier course content, and the specific client case remained memorable. Notes prepared for the CBA were perceived as valuable for future clinical practice.

“I’m not sure I learned anything new, but it was a good recap of what we went through.” (Student 14)

“Now I have notes ready for similar situations. If I’m working in outpatient care or somewhere with frequent clients, I can quickly check my notes before going in.” (Student 13)

### Supporting professional growth

The CBA allowed students to reflect on their professional identity and their relationship to physiotherapy work. Many felt that the experience strengthened their professional development, especially in terms of confidence and self-assurance. Success in the CBA increased trust in their own abilities and reinforced the belief that they could manage in real work settings. However, some also considered how failure might negatively affect their confidence.

“If you can handle something like this, you’ll probably manage in working life too.” (Student 16)

“It boosted my confidence. I didn’t prepare that much, and it went well. It made me feel like working life won’t be much harder.” (Student 27)

Students also reflected on the importance of client interaction. The CBA provided a chance to test essential aspects of physiotherapy, especially communication skills, which hadn’t been assessed in previous exams. Many emphasized that genuine presence and connection with the client were more important than perfect technical performance. They also noted that physiotherapists don’t need to know everything immediately, revisiting and reviewing is part of the process. The CBA reinforced the idea that physiotherapists can work in a personal and authentic way, as long as their actions are grounded in professional principles and goals. Discussions also touched on the potential conflict between client expectations and physiotherapy goals, and how to navigate such situations professionally.

“It really tests the most important areas… you might not know everything, but it also checks whether you can connect with the patient.” (Student 3)

“It strengthened the idea that meeting the client is the most important part and being present in the situation. And that physiotherapy can be done in your own style, as long as you’re doing the right things.” (Student 2)

“It reinforced the idea that you shouldn’t see the client only through their shoulder pain. They’re a person, with a family, job, goals, and dreams. The experience tied it all together: the shoulder might be the reason they came, but it’s really about the person in their own context.” (Student 1)

## Discussion

This qualitative study explored physiotherapy students’ experiences of the CBA at University of Applied Sciences. Thematic analysis revealed three main themes: the emotional experience of CBA, its role in enabling the learning process, and its perceived usefulness for developing future professional competencies. Overall, students’ experiences were predominantly positive. The CBA was perceived as a realistic and well-structured simulation that supported both competence evaluation and learning.

Overall, students responded positively to the CBA, aligning with previous findings.^7,8,10,11^ Many reported experiencing significant stress and nervousness beforehand, which eased once the exam began.^14^ Unlike in some international studies,^10,12,13,19^ the CBA situation in this study was perceived as relaxed and supportive, suggesting successful creation of a safe learning environment.

Students prepared for the CBA in diverse ways, with varying levels of effort. Uncertainty about what to focus on due to broad subject areas was a common challenge, consistent with previous research.^13,17^ The post-assessment evaluation discussion was highlighted as a meaningful learning opportunity, helping students reflect on their performance. This practice appears to be more emphasized in Finland than internationally.^14^

Similar approaches to CBA have been reported across other health professions internationally, although implementation practices and the emphasis on authenticity vary between countries.^15,17^ In line with these broader findings,^7,8,14,15,17^ in our context teacher performance and the authenticity of the CBA environment played a key role in shaping student experiences. While most students appreciated the teacher’s role-play as a client, some questioned the realism of the situation. These reflections mirror findings from other studies using actors^20^ or dual-role teachers.^15^ These differing perceptions may also reflect variation in students’ prior clinical experience and their comfort with role-play, factors that have been shown to influence how authentic and meaningful simulated encounters are perceived to be.^15,17,20^ At the same time, when the teacher acted as the simulated client, they were able to observe the student’s practical skills and provide targeted, clinically relevant feedback, such as on manual techniques or patient guidance, which students considered beneficial. Evidence from OSCE-based assessments likewise highlights the importance of clear examiner facilitation and constructive feedback in supporting learning and maintaining the purposefulness of the assessment encounter.^14,17^

Students found the CBA useful for realistically evaluating their competence and appreciated the feedback, which was seen as beneficial not only for themselves but also for educators in understanding students’ skill levels. Some students expressed a desire for numerical grading, believing it could enhance motivation to prepare for the CBA. Concerns were raised about the fairness of different client cases, with students reflecting on whether all scenarios were equally challenging and how they might have performed in a different situation. These reflections align with previous studies, which have noted that although CBA are generally perceived as fair and effective tools for evaluating skills, students have raised concerns about task balance when difficulty levels vary.^8,10,14,17^

Findings from this study suggest that physiotherapy students perceived the CBA as supportive of both professional growth and readiness for working life. The CBA increased their confidence in their skills and strengthened their belief in their ability to work in the field. It provided an opportunity to apply theoretical knowledge in a practical, clinical context. While not all students felt they learned something new during the CBA itself, the value of revisiting and consolidating prior learning was widely recognized, consistent with previous research.^8^

Our findings on increased professional confidence also align with physiotherapy-specific literature. Hlebš (2021) found that graduating physiotherapy students rated their overall competence higher in clinical settings than in the university context, as authentic clinical environments made the complexity of real practice more visible and helped students more accurately judge their abilities.^21^ The authentic structure of the CBA may similarly support students in recognizing and strengthening their professional competence by providing realistic scenarios in which theoretical knowledge must be applied in practice.

Similarly, students’ perceptions of improved work readiness echo the transition challenges described by Stoikov et al (2022), who highlighted the difficulties new graduates face in managing clinical complexity.^22^ Through engagement with realistic scenarios requiring prioritization and problem-solving, the CBA may help familiarize students with the cognitive and practical demands typical of early professional practice.

In summary, CBA appear to enhance the quality of physiotherapy education. They integrate problem-solving, clinical reasoning, and interpersonal skills, which are core elements of professional practice, and offer students a valuable opportunity to develop and demonstrate their competence in authentic scenarios.

## Strengths, Limitations, and Future Directions for Research

The study benefited from a relatively large number of participants and the inclusion of multiple small groups, which enriched the diversity of perspectives. Data were collected the day after the CBA, at a point when the experience was still fresh, but the initial emotional intensity had subsided. Furthermore, the study focused on a student group that has received relatively little research attention, adding relevance and novelty to the findings. The initial coding was conducted by one researcher, after which the entire research team collaboratively reviewed and refined themes to ensure credibility and trustworthiness. The interviewer’s physiotherapy background was an additional strength, as it supported contextual understanding and helped create a comfortable and trusting atmosphere during interviews. At the same time, the interviewer remained aware that her professional background and possible preconceptions could influence data collection and interpretation, and she actively sought to minimize these effects through reflexive practice.

Although this study was carefully conducted and the data were described transparently, some limitations must be acknowledged. These study findings are transferable to other Universities of Applied Sciences in Finland that provide competence-based physiotherapy education for practice within the Finnish healthcare system across both the public and private sectors. The results are also applicable to European bachelor-level physiotherapy programmes that operate within comparable educational structures and principles. However, the research focused on a single group of physiotherapy students, which may limit the transferability of the findings. While efforts were made to describe the CBA context and environment in detail, the results may not fully represent broader student populations or other educational settings. Moreover, certain features of CBA design may have influenced students’ experiences. The predetermined 60% passing threshold may have shaped students’ perceptions of assessment related performance pressure and the level of preparation required, while the use of teachers as simulated clients, although practical and standardized, may have reduced the perceived authenticity of the assessment situation. Moreover, CBA should be understood as one component within the broader physiotherapy curriculum. To support students’ readiness, the assessment elements should be practiced earlier in the programme, for example through simulation-based learning activities. Ensuring that the assessment cases reflect a comparable level of complexity is also essential for maintaining consistency and fairness across student evaluations.

Group interviews provided rich discussions but may have influenced participants’ willingness to share differing or personal views. Some students participated less actively, which may have affected the depth and diversity of the data. Individual interviews could have offered additional perspectives.

Future research could explore educators’ perspectives on organizing and assessing CBA. It would be valuable to examine how educators balance authenticity and support when designing CBA, and how these design choices influence the implementation of the assessment. It would also be worthwhile investigating students’ views on the relevance and impact of CBA after entering working life. Such studies could be conducted retrospectively or through longitudinal designs following students across different stages of their professional development.

## Conclusions

This study provides initial insights into physiotherapy students’ experiences of a newly piloted CBA in Finland. The findings suggest that the CBA offers a valuable opportunity to evaluate graduating students’ clinical competence in a realistic and supportive environment. The simulation-based format was perceived to strengthen learning and professional confidence, especially through authentic practice and post-assessment evaluation discussions. While students commonly experienced pre-assessment nervousness, a calm and well-structured atmosphere helped ease anxiety and enhance the learning experience. These results support the integration of CBA into physiotherapy education as a meaningful way to prepare students for working life. In particular, the findings point to practical considerations for educators, such as providing clear pre-assessment guidance to reduce anxiety, ensuring authenticity in teacher role-plays, and allocating sufficient time for high-quality post-assessment feedback discussions to optimize learning.

## Data Availability

The data that supports the findings of this study are available from the corresponding author upon reasonable request.*
